# Dendritiform immune cells with reduced antigen-capture capacity persist in the cornea during the asymptomatic phase of allergic conjunctivitis

**DOI:** 10.1038/s41433-023-02413-2

**Published:** 2023-02-06

**Authors:** Zahra Tajbakhsh, Isabelle Jalbert, Fiona Stapleton, Ali Alghamdi, Paul E. Gray, Nancy Briggs, Betina Altavilla, Rabia Mobeen, Blanka Golebiowski

**Affiliations:** 1 School of Optometry and Vision Science, UNSW, Sydney, Australia; 2 Department of Immunology and Infectious Diseases, Sydney Children’s Hospital, Sydney, Australia; 3 Stats Central, Mark Wainwright Analytical Centre, UNSW, Sydney, Australia

**Keywords:** Eye diseases, Outcomes research

## Abstract

**Background:**

Increased density and altered morphology of dendritic cells (DC) in the cornea and conjunctiva occur during active allergic conjunctivitis. This study investigated whether inflammation (characterised by altered DC density and morphology) persists during the symptom-free phase of allergic conjunctivitis.

**Methods:**

Twenty participants (age 43.3 ± 14.3 years, 55% female) assessed during their active (symptomatic) phase of allergic conjunctivitis were re-examined during the asymptomatic phase. Ocular allergy symptoms and signs were evaluated during both phases, and five ocular surface locations (corneal centre, inferior whorl, corneal periphery, corneal limbus, and bulbar conjunctiva) were examined using in vivo confocal microscopy (HRT III). DC were counted manually, and their morphology was assessed for cell body size, presence of dendrites, presence of long dendrites and presence of thick dendrites using a grading system. Mixed model analysis (DC density) and non-parametric tests (DC morphology) were used to examine differences between phases.

**Results:**

DC density at corneal locations did not change between the active and asymptomatic phases (*p* ≥ 0.22). However, corneal DC body size was smaller and fewer DC presented with long dendrites during the asymptomatic phase (*p* ≤ 0.02). In contrast, at the bulbar conjunctiva, DC density was reduced during the asymptomatic phase compared to the active phase (*p* = 0.01), but there were no changes in DC morphology.

**Conclusions:**

Dendritiform immune cell numbers persist in the cornea during the symptom-free phase of allergic conjunctivitis, whereas conjunctival DC appear to return to a baseline state. The morphology of these persisting corneal DC suggests their antigen-capture capacity is reduced during the asymptomatic phase.

## Introduction

Dendritic cells (DC) are a subtype of immune cells which play a pivotal role in the ocular surface defence system, and the initiation of the immune response in allergic conjunctivitis [1–3]. A higher density and altered morphology of DC has been shown in the cornea and conjunctiva during the active, symptomatic phase of allergic conjunctivitis using in vivo confocal microscopy (IVCM) [4]. In severe forms of ocular allergy including vernal keratoconjunctivitis (VKC), DC have been described as hyperreflective cells with dendritic processes and their density was higher at the corneal and conjunctivital locations compared to the healthy group [5–7].

Allergic conjunctivitis has been described as an allergen-induced inflammatory reaction that results in bothersome symptoms and signs, reduced quality of life, and carries a significant societal burden [8, 9]. The hallmarks of allergic conjunctivitis include bulbar hyperaemia and itching, and current ocular allergy treatments are titrated based on symptoms and signs [10].

Persistent low-grade inflammation (ICAM-1/CD54 expression) has been detected in the conjunctival and nasal epithelium of rhinitis during asymptomatic periods [11, 12]. In allergic rhinitis, subthreshold doses of allergens can cause inflammatory cell infiltration at the mucosal level in the absence of allergy symptoms [12, 13]. Persistent inflammatory infiltrates in the respiratory epithelium of asthma patients have been associated with increased susceptibility to viral infections [12]. It has been suggested that as allergic rhinitis patients with minimal persistent inflammation are at increased risk for developing allergy symptoms, anti-inflammatory treatment should continue during their symptom-free period [11]. This therapeutic approach in patients with allergic rhinitis considers symptoms as the ‘’tip of the iceberg” with inflammation and hyperresponsiveness “below” or in the submerged part of the iceberg [14].

Likewise, consideration of the baseline inflammatory state of allergic conjunctivitis patients may help to inform and thus achieve better outcomes for the management of their allergic eye disease. A recent study reported higher DC density, longer dendrites, and a larger cell field at the corneal centre during the quiescent phase of VKC, when compared to a healthy control group, indicating an ongoing subclinical inflammatory process during the quiescent phase [15]. Overexpression of proinflammatory tear proteins was greater in VKC patients during both active and quiescent phases compared to a group of non-allergic patients, also suggesting ongoing inflammation during the quiescent phase [16]. It is not known whether a similar sub-clinical inflammation remains in the asymptomatic phase of the more prevalent form of ocular allergy, allergic conjunctivitis.

In the present study, we investigated whether inflammation (characterised by altered DC density and morphology) persists during the symptom-free phase of allergic conjunctivitis.

## Methods

### Study design and participants

A prospective, observational, two visit study was conducted. The participants were a subgroup from a larger cross-sectional study involving 33 participants with allergic conjunctivitis and 33 healthy controls, the results of which have been reported elsewhere [4]. Twenty allergic participants who responded to the invitation to attend a second visit study were included in the current study.

Ethics approval was obtained from the Sydney Children’s Hospital Network Human Research Ethics Committee (HREC) (2019/ETH11844) and HREC of UNSW Sydney (HC180930). The study followed the Declaration of Helsinki, and informed consent was obtained from all participants.

Participants were included who were aged over 18 years, with or without a prior diagnosis of allergic conjunctivitis, who had current symptoms of ocular allergy or hay fever, and a positive skin prick test. Standard skin prick test for ten common indoor and outdoor aeroallergens in the Sydney region was conducted (Stallergenes, Antony, France, and Inmunotek, alergie e immunologia, Spain): three grass pollens, tree pollen, plant pollen, two dust mites, mould, cat, and dog dander. The skin prick test was considered positive for an allergen wheal size ≥ 3 mm [17].

Exclusion criteria included: current use of topical or systemic antihistamine/mast cell stabilizer/nasal corticosteroid sprays, use of lubricants in the week before the study, current immunotherapy for aeroallergens, uncontrolled asthma, past anaphylactic episode, current pregnancy or breastfeeding, regular contact lens wear, any current ocular surface disease other than ocular allergy, Sjøgren syndrome, active intraocular inflammation, history of corneal refractive surgery/ocular surgery, or systemic conditions that can affect the ocular surface (e.g., diabetes, rheumatoid arthritis).

Sample size was calculated using GPower 3.1 based on finding a mean difference equal to or greater than the coefficient of repeatability for DC density at the corneal centre [18]. A sample size of 20 participants would have greater than 80% power to detect a mean difference of 28 cell/mm^2^ for DC density at the corneal centre (SD = 35 cells/mm^2^) [18], including a 15% adjustment to allow for possible non-normal distribution in outcome variable [19].

The first study visit (“active phase”) was conducted during September 2019 to January 2020 [20], when participants were experiencing symptoms of allergic conjunctivitis. The second study visit (“asymptomatic phase”) was conducted during July to August 2020 and when participants had reported at least four symptom free weeks. Assessments were conducted at both visits, as outlined below.

### Ocular surface symptoms and signs

Participants completed symptoms questionnaires related to ocular allergy (Aston University Allergy Questionnaire, AUAQ) [21] and dry eye (Ocular Surface Disease Index, OSDI, Dry Eye Questionnaire, DEQ-5) [22, 23]. Non-invasive tear break-up time was assessed using the hand-held tearscope (Keeler Ltd, United Kingdom). A grid attachment was used to form a reflection from the tear film. The time taken for the first break to appear on the tear film (distorted grid pattern) was recorded using the timer on the tearscope. Ocular surface redness was graded using the BHVI grading system [24] corneal epithelial disorders, conjunctival chemosis, papillae, and follicles were graded using the Japanese grading scale [25]. The Oxford grading scale was used to grade ocular surface staining [26].

### In vivo assessment of DC density and morphology using confocal microscopy

An HRT III confocal microscope with a Rostock Corneal Module (Heidelberg Engineering GmbH, Heidelberg, Germany) was used to capture images of corneal and conjunctival epithelial DC in vivo from the right eye. Five locations of the cornea and conjunctiva were scanned in the following order: corneal anatomical centre, corneal inferior whorl (densely innervated area located in the inferonasal cornea), far peripheral cornea (temporal, 1 mm inside limbus), limbal cornea (temporal), and bulbar conjunctiva (temporal, 2–3 mm from the limbus). The corneal sub-basal epithelium was detected at approximately 35–70 µm depth and the conjunctival epithelium at 5–20 µm depth.

#### Image analysis

Five best-focused images overlapping by less than 20% were selected from each corneal and conjunctival location; only 1 image from the inferior whorl was included due to the small area. Images were assessed for DC density and morphology by an experienced investigator masked to the study phase (active or asymptomatic) and image location.

#### DC density

Bright hyperreflective cells of at least 10 µm in size, with a linear/ curvilinear cell body, with or without dendrites (both short and long) located at the sub-basal corneal epithelium and distributed among nerve fibres that do or do not cross them, or at the conjunctival epithelium, were considered as DC [27]. DC density was counted manually and the mean value of 5 images recorded as cells/mm^2^ other than for the inferior whorl where the value for a single image was recorded.

#### DC morphology

For DC morphology, cell body size and presence of dendrites at all locations were assessed using a grading system [27]. Briefly, cell body size was graded as small (10–25 µm), medium (26–40 µm), or large (> 40 µm), based on the largest cell body size observed in any of the 5 images. The presence of any dendrites, the presence of long dendrites and the presence of thick dendrites in any of the 5 images was recorded. In addition, the presentation of DC in clusters (wire netting pattern) in the conjunctival epithelium was noted [5].

### Statistical analysis

To examine differences in symptoms and signs between the two phases, the Wilcoxon Signed Ranks Test or Paired Sample t-test were used.

DC density was not normally distributed and therefore DC density values were log-transformed after adding 0.5 to density values of zero before entering into the mixed model. A linear mixed model with a random effect for individuals, and fixed effects of study phase and ocular surface location was used to examine differences in DC density between phases.

Wilcoxon Signed Ranks test (for cell body size) and McNemar test (for presence of dendrites) were used to assess differences in DC morphology between phases, at each location.

In addition, DC density and morphology during the asymptomatic phase was compared to previously reported results for 33 healthy non-allergic participants [4]. A linear mixed model with a random effect for individuals and fixed effect of group and ocular surface location was used to examine differences in DC density between groups. Mann- Whitney U test (for cell body size) and Fisher’s Exact Test (for presence of dendrites) were used to assess differences in DC morphology between groups, at each location.

Associations between DC and ocular surface symptoms and signs were initially assessed using univariate Spearman’s correlation (for DC density and cell body size) and Mann-Whitney U test (for presence of dendrites). Symptoms and signs that were significant at *p* < 0.01 (adjusted for multiple comparisons) were added to a multivariate generalised estimating equation. A backward elimination modelling approach was used, and the final model included only those signs and symptoms that were significantly associated with DC density or morphology at *p* < 0.05. Associations between significant changes in DC density between phases, and changes in symptoms and signs (for continuous variables) which occurred over the same time frame, were examined using Spearman’s correlation.

*P* < 0.05 was considered statistically significant.

## Results

Twenty participants (mean age 43.3 ± 14.3 years, 55% female) completed the study. All participants tested positive to at least one of the dust mites or pollen allergens in the skin prick test; 80% tested positive to dust mites, 85% to at least one type of pollen, and 65% were positive to both dust mites and pollen (Supplementary Table 1).

### Clinical findings

Ocular surface symptoms and signs in both phases (active and asymptomatic) are shown in Table 1. More ocular allergy symptoms were reported in the active phase of allergy with the AUAQ (*p* ≤ 0.04). Participants also reported more dry eye symptoms in the active phase with the DEQ-5 (*p* = 0.04). During the active phase, participants had higher limbal, bulbar, and palpebral redness, conjunctival chemosis, follicles, and conjunctival staining (*p* ≤ 0.01), and a lower non-invasive tear break-up time (*p* = 0.03).

**Table 1:** Summary of findings for ocular surface symptoms and signs in allergic participants during the active (*n* = 20) and asymptomatic (*n* = 20) phases of allergy.

Variable		Active phase *n* = 20	Asymptomatic phase *n* = 20	*p*-value
Ocular surface symptoms	AUAQ, Total symptom score (0–21)	7 (3–18)	3 (0–10)	***<0.001***
	Dryness (0–3)	1 (0–2)	1 (0–2)	***0.04***
	Itchiness (0–3)	1 (0–3)	0 (0–2)	<***0.001***
	Burning (0–3)	0 (0–3)	0 (0–1)	***0.03***
	Stinging (0–3)	0 (0–3)	0 (0–1)	0.08
	Watering (0–3)	1 (0–3)	0 (0–2)	0.3
	Redness (0–3)	1 (0–2)	0 (0–2)	0.08
	A need to rub eyes (0–3)	2 (0–3)	1 (0–2)	***0.01***
	OSDI (0–100)	16.6 (2.0–45.8)	14.5 (0–37.5)	0.06
	DEQ–5 (0–22)	9.0 (1.0–16.0)	7.0 (0–17.0)	***0.04***
Ocular surface signs	Limbal redness (0–4, 0.1)	3.0 (1.5–4.0)	1.0 (0.5–2.5)	***<0.001***
	Bulbar redness (0–4, 0.1)	2.4 (1.7–3.5)	1.0 (0.50–2.1)	***<0.001***
	Palpebral redness (0–4, 0.1)	2.5 (2.0–3.5)	1.0 (0.5–2.0)	***<0.001***
	Corneal epithelial disorder (0–3, 1)	0 (0–0)	0 (0–0)	1.0
	Bulbar conjunctival chemosis (0–3, 1)	1 (0–2)	0 (0–1)	***<0.001***
	Palpebral conjunctival papillae (0–3, 1)	0 (0–0)	0 (0–0)	1.0
	Palpebral conjunctival follicle (0–3, 1)	0 (0–2)	0 (0–0)	***0.01***
	Corneal staining (0–5, 1)	0 (0–0)	0 (0-0)	1.0
	Conjunctival staining-Nasal (0-5, 1)	0 (0–2)	0 (0–1)	***0.004***
	Conjunctival staining-Temporal (0–5, 1)	1 (0–2)	0 (0–1)	***0.002***
	Non-invasive Tear film Break-Up Time (seconds)	9.5 (5.8–17.5)	12.5 (5.8–24.0)	***0.03***

Ocular signs are graded in integers except for limbal, bulbar, and palpebral redness which were graded in 0.1 steps. Statistically significant values (*p* < 0.05) are indicated in bold/italics. *AUAQ* Aston University Allergy Questionnaire, *OSDI* Ocular Surface Disease Index, *DEQ-5* Dry Eye Questionnaire.

Values are reported in median (range).

### DC density

**Fig. 1:**
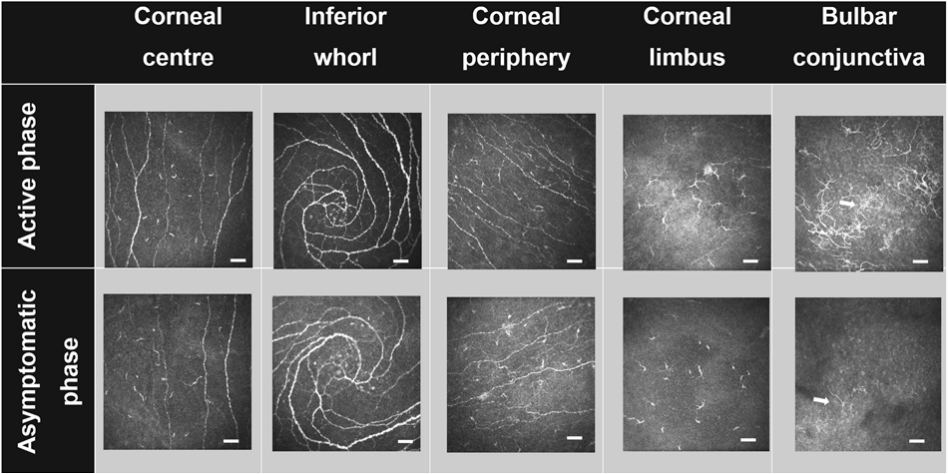
Representative IVCM images of corneal sub-basal and conjunctival epithelium in the active (top row) and asymptomatic (bottom row) phase of allergic conjunctivitis. Arrows indicate DC at bulbar conjunctiva, that were higher in density during the active phase of allergy. Bulbar conjunctival DC in the active phase tended to gather in cluster (top right). Image size = 400 × 400 µm; bar = 50 µm.

Representative IVCM images at each ocular surface location are shown in Fig. 1.

**Fig. 2:**
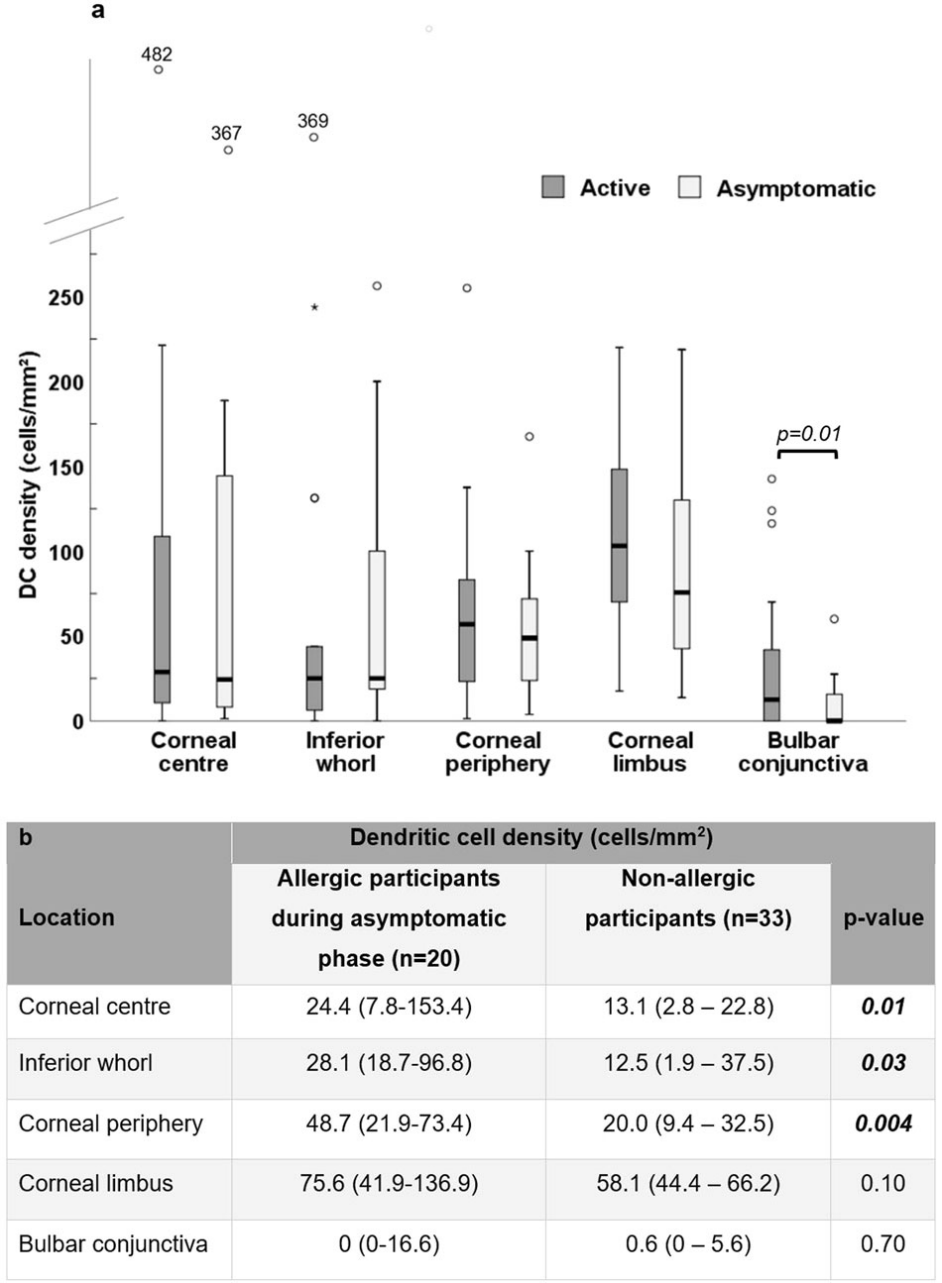
Dendritic cell density at various corneal and conjunctival locations. Dendritic cell (DC) density across corneal and conjunctival locations in participants during active (*n* = 20) and asymptomatic (*n* = 20) phases of allergic conjunctivitis (**a**), and during the asymptomatic phase of allergic conjunctivitis (*n* = 20), and in non-allergic participants (*n* = 33) (**b**). Plots represent median (horizontal black line), interquartile range (box), lower and upper extremes (whiskers) and outliers lying above Q3 + 1.5*interquartile range and below Q1-1.5*interquartile range (circles). Data in the table are expressed as median (IQR). Data for the non-allergic participants are from a previous study, reported elsewhere [4].

The fixed effect of phase was not significant (*F* = 0.82, *p* = 0.36) showing that overall, there were no significant differences in DC density between phases. Simple main effects showed an effect of phase only at the conjunctiva showing that DC density was elevated in the active phase compared to the asymptomatic phase (*p* = 0.01) (Fig. 2a, Supplementary Table 2).

DC density was significantly different between allergic participants during the asymptomatic phase and a group of non-allergic participants (*F* = 10.2, *p* = 0.002). Simple main effects showed that DC density was higher in allergic participants during the asymptomatic phase of allergy compared to the non-allergic group at the corneal centre (*p* = 0.01), inferior whorl (*p* = 0.03) and corneal periphery (*p* = 0.004) but was not significantly different at the limbus or conjunctiva (Fig. 2b).

### DC morphology

**Fig. 3:**
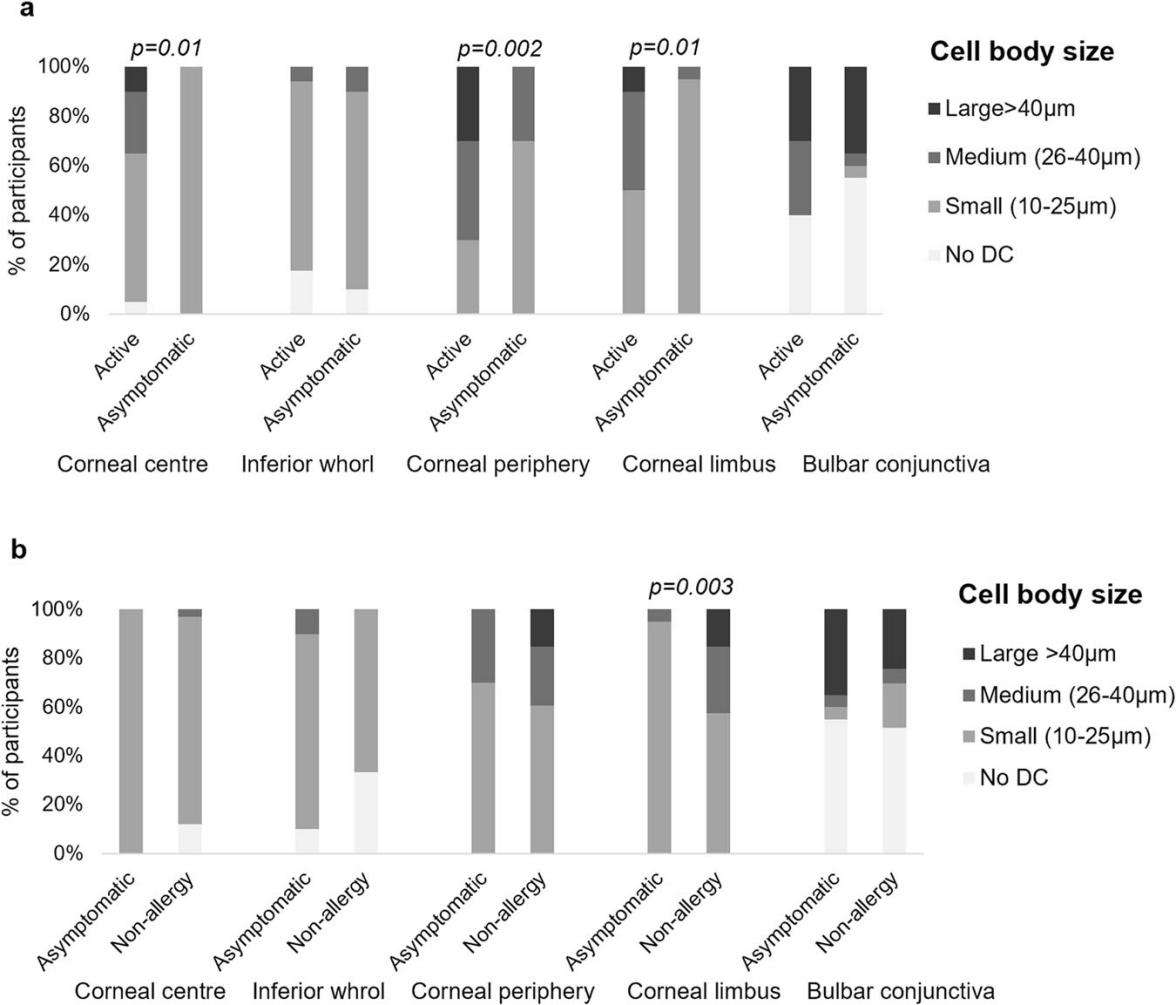
Dendritic cell body size at various corneal and conjunctival locations. Dendritic cell (DC) body size across corneal and conjunctival locations during the active (*n* = 20) and asymptomatic (*n* = 20) phase of allergic conjunctivitis (**a**), and during asymptomatic phase (*n* = 20) of allergic conjunctivitis and non-allergic control group (*n* = 33) (**b**). Images devoid of dendritic cells were excluded. Data for the non-allergic participants are from a previous study, reported elsewhere [4].

DC body size at the corneal centre, peripheral cornea and limbus was smaller during the asymptomatic phase than the active phase of allergic conjunctivitis (*p* ≤ 0.01), but not in the conjunctiva (*p* = 1.0) (Fig. 3a).

The percentage of DC presenting with dendrites, with long dendrites and thick dendrites was reduced during the asymptomatic phase of allergy at almost all corneal locations, however, these differences were only significant for the presence of long dendrites at the corneal centre and limbus (*p* ≤ 0.02) (Table 2a).

**Table 2:** Corneal and conjunctival dendritic cell morphology for dendrite appearance (presence of dendrites, presence of long and thick dendrites) in active (*n* = 20) and asymptomatic (*n* = 20) phases of allergic conjunctivitis (a) and in asymptomatic phase of allergic conjunctivitis (*n* = 20), and non-allergic control group (*n* = 33) (b).

a	Presence of dendrites			Presence of long dendrites			Presence of thick dendrites		
	Active *n* = 20	Asymptomatic *n* = 20	*p*-value	Active *n* = 20	Asymptomatic *n* = 20	*p*-value	Active *n* = 20	Asymptomatic *n* = 20	*p*-value
Corneal centre	63%	45%	0.45	42%	5%	***0.02***	11%	5%	1.0
Inferior whorl	36%	17%	0.50	21%	0	0.25	0	0	NA
Corneal periphery	95%	90%	1.0	65%	35%	0.07	15%	5%	0.60
Corneal limbus	100%	75%	0.06	80%	35%	***0.01***	25%	5%	0.20
Bulbar conjunctiva	100%	100%	NA	92%	100%	1.0	0	11%	1.0

b									
	Asymptomatic *n* = 20	Non-allergy *n* = 33	*p*-value	Asymptomatic *n* = 20	Non-allergy *n* = 33	*p*-value	Asymptomatic *n* = 20	Non-allergy *n* = 33	*p*-value
Corneal centre	45%	28%	0.25	5%	7%	1.0	5%	0	0.40
Inferior whorl	17%	9%	0.65	0	0	NA	0	0	NA
Corneal periphery	90%	79%	0.45	35%	49%	0.40	5%	3%	1.0
Corneal limbus	75%	97%	***0.02***	35%	73%	***0.01***	5%	15%	0.40
Bulbar conjunctiva	100%	94%	1.0	100%	94%	1.0	11%	0	0.35

Images devoid of dendritic cells were excluded and thus data are presented for participants with dendritic cells at each location. Data for the non-allergic participants are from a previous study, reported elsewhere [4].

Statistically significant values (*p* < 0.05) are indicated in bold/italics.

In the conjunctiva, DC tended to gather in a cluster with wire netting pattern in 58% of participants during the active phase, compared to 22% in the asymptomatic phase (Fig. 1).

DC morphology was significantly different in allergic participants during the asymptomatic phase compared to non-allergic participants at the corneal limbus only. During the asymptomatic phase of allergy, limbal DC bodies were smaller (*p* = 0.003), and a lower proportion of limbal DC presented with dendrites and with long dendrites (*p* ≤ 0.01), than in non-allergic participants. There were no differences between groups in DC morphology at the corneal centre, periphery, inferior whorl or conjunctiva (*p* ≥ 0.41), Fig. 3b and Table 2b).

### Associations between DC density and morphology and ocular surface symptoms and signs

Weak associations were observed in univariate analysis between DC body size and limbal, bulbar and palpebral redness, and conjunctival staining (rho ≤ 0.27, *p* < 0.01) and between presence of long dendrites and limbal, bulbar and palpebral redness (*p* = 0.001) (Supplementary Table 3). No significant associations between DC body size and presence of long dendrites and any of these signs were evident in the multivariate model.

No associations were found between DC density, presence of dendrites and presence of thick dendrites, and ocular surface symptoms and signs.

The decrease in DC density in the conjunctiva between the active and asymptomatic phase was not associated with the changes in symptoms or signs which occurred over the same time frame (−0.30 ≥ rho ≤ 0.29, *p* ≥ 0.32), supplementary table 4.

## Discussion

Corneal and conjunctival epithelial DC density and morphology were assessed twice in vivo in allergic patients, during both active and asymptomatic phases of allergic conjunctivitis. DC density at the bulbar conjunctiva was decreased during the asymptomatic phase compared to the active phase, but corneal DC density remained elevated in both phases. In contrast, DC morphology at the cornea changed between phases but remained unaltered at the conjunctiva.

Inflammation in the cornea persisted during the asymptomatic phase of allergic conjunctivitis. DC density in the cornea of allergic participants during the asymptomatic phase was higher than in a comparative non-allergic group. This confirms the presence of ongoing corneal inflammation during the asymptomatic phase of allergic conjunctivitis. Similarly, subclinical inflammation at the corneal centre during quiescent VKC has previously been reported in an IVCM study showing higher DC density in VKC patients (44.03 ± 32.86 cells/mm^2^) compared to a non-allergic group (20.68 ± 13.69 cells/mm^2^) [15].

DC density at all corneal locations remained elevated during the asymptomatic phase of allergy and was higher than in a comparative non-allergic group at all corneal regions other than the limbus. Comparison of limbal DC density during the asymptomatic phase with healthy non-allergic participants showed that limbal DC density during the asymptomatic phase had returned to normal (non-allergic) levels in most but not all participants.

These data suggest that some limbal DC may behave similarly to conjunctival DC, due to their relative proximity to limbal lymphatic vessels which may facilitate their migration toward the draining lymph nodes [28, 29]. DC recruited from the blood to the limbus in the presence of inflammation can increase limbal DC density which then migrate toward the centre [30, 31]. In an experimental mice model, intravenously injected bone marrow derived antigen presenting cells were found to reach limbus first and then migrated centripetally [32].

This study was the first to examine DC density and morphology as a marker of allergic immune response in participants during both active and asymptomatic phases of allergic conjunctivitis. Allergic participants had mild to moderate symptoms and clinical signs of allergic conjunctivitis which significantly decreased during the asymptomatic phase. Symptoms are a unique marker of allergic disease [10], and allergy therapeutic decision-making remains primarily symptoms-guided [33]. Confirmation of the presence of inflammation during the symptom-free period in several allergic diseases is beginning to challenge this paradigm. The findings from this study suggest that DC density and morphology may be more sensitive markers of inflammation than allergic symptoms and other clinical signs and should perhaps be considered in the therapeutic decision-making for allergic eye diseases. As there was no association between DC density, morphology, and the ocular allergy symptoms/ signs, nor between the changes in conjunctival DC and changes in symptoms/ signs which occurred over the same time frame, this might suggest that in mild and moderate state of the allergic conjunctivitis DC is a more robust marker of inflammation compared to the clinical presentation, this warrants further evaluation in ocular allergy of various severities.

Low level persistent inflammation in asymptomatic allergy patients may exacerbate allergic reactions during periods of acute allergen increase. In allergic rhinitis patients who were clinically in the asymptomatic phase, the presence of such minimal persistent inflammation was related to a smaller dose of allergens needed to provoke allergy symptoms [11, 34]. Avoidance of allergens such as pollens and dust mites may not always be feasible and thus a preventative approach which involves prophylactic treatment of allergic patients during their asymptomatic phase could perhaps be warranted in allergic conjunctivitis. Studies in allergic rhinitis patients reported prophylactic treatment during asymptomatic periods to be effective in delaying the onset and reducing progression and severity of the disease [11, 35, 36].

The findings of this study suggest that the conjunctiva returns to a non-inflamed state more rapidly than the cornea, in allergic conjunctivitis. DC density at the conjunctival epithelium decreased during the asymptomatic phase of allergic conjunctivitis compared to the active phase, to a level comparable to that of a healthy non-allergic group. In contrast, a subclinical inflammatory state was observed during the symptom-free period in the nasal and conjunctival epithelium in patients with allergic rhinitis who were allergic to dust mites and in the nasal epithelium of allergic rhinitis patients allergic to pollens [12, 37]. Expression of intercellular adhesion molecule 1 (ICAM-1/CD54), and the presence of eosinophils and neutrophils at the conjunctival and nasal mucosa was detected in the absence of allergy symptoms in these patients [12, 37, 38]. The more rapid return of conjunctival versus corneal DC density to a baseline state could be explained by the presence of lymphatic vessels in the conjunctiva but not the cornea. Conjunctival lymphatic vessels may have a role in the trafficking of DC in the conjunctiva including the elimination of DC from conjunctival tissue [2, 39]. Conjunctival DC morphology did not differ between the active and asymptomatic phases nor with the non-allergic group. However, in more than half of the participants during the active phase and a quarter in the asymptomatic phase, conjunctival DC presented in a wire netting pattern, similar to that previously reported in the conjunctiva of VKC patients [5]. This appearance was not observed in the non-allergic group [4]. This suggests a possible pattern-specific inflammatory response at the conjunctiva and might be representative of a higher degree of response during the active phase of allergy.

Corneal DC morphology was altered between the active and asymptomatic phases of allergy, displaying a smaller cell body size and fewer dendrites. DC bodies and presence of dendrites did not differ significantly during the asymptomatic phase compared to the non-allergy group other than limbal DC which displayed smaller cell bodies, and fewer dendrites and long dendrites. The presence of fewer long dendrites is likely to indicate a less activated DC state with reduced antigen capture capacity whereas fewer thick dendrites may be associated with reduced migration capacity [27]. In contrast, during the quiescent phase of VKC, corneal DC were observed to have longer dendrites and a larger cell field compared to a non-allergic group [15]. These differences may reflect differences in the severity and pathophysiology of allergic conjunctivitis and VKC [40]. Upon activation of the immune system, several DC changes occur, including changes in the size of cell body along with changes in the DC dendrite appearance including length, thickness and number of dendrites [41–46]. We have previously reported larger DC bodies and a greater proportion of DC with dendrites and with long dendrites in patients with active allergic conjunctivitis compared to a non-allergy group [4]. We have also shown DC with longer dendrites in patients with systemic allergy with and without ocular allergy symptoms [47]. The in vivo assessments of DC activation described in this study are based on morphology without additional information regarding specific DC activation markers. However, immunohistochemistry studies have previously correlated morphology of DC with expression of activation markers such as HLA-DR and MHC class II. These studies showed that DC with short dendrites have less capacity for antigen capture and are mainly present in healthy eyes, whereas DC with long dendrites are positive for activation markers and are mainly present in inflamed eyes with enhanced antigen capturing capacity [48, 49]. Our data support the use of morphology as a possible and accessible biomarker in allergic eye diseases.

Although IVCM has the distinct benefit of allowing imaging of living tissue, it is not able to confirm cell phenotype and provide critical information about cell surface markers required to confirm the cells observed are in fact dendritic cells. Use of other methods including immunohistochemistry to cross-validate the results of IVCM would be of benefit in the conjunctiva but is not possible in human corneal tissue.

In conclusion, this study showed the presence of persistent inflammation in the cornea but not in the conjunctiva of participants with allergic conjunctivitis during the symptom-free period. Inflammation may resolve more promptly at the limbus compared to other corneal locations. The morphology of these persistent DC (smaller cell body size, fewer long dendrites) suggests their antigen-capture capacity was reduced during this asymptomatic phase. This work lays the foundations for further studies of ocular allergy of different severities, and to explore the usefulness of DC density, morphology, and topographical distribution as a marker of efficacy of allergy treatment.

## Summary

### What was known before

- A higher corneal and conjunctival dendritic cell density and altered morphology have been reported during active allergic conjunctivitis.
- Persistent low-grade inflammation has been detected in the conjunctival and nasal epithelium of rhinitis patients in the absence of allergy symptoms.

### What this study adds

- This study confirmed the presence of ongoing inflammation in the cornea but not in the conjunctiva of allergic conjunctivitis sufferers during the symptom-free period.
- DC density and morphology may be a more sensitive marker of inflammation than allergic symptoms and signs and should be considered in the management of allergic eye diseases.

## Supplementary information

Supplementary table 1

Supplementary table 2

Supplementary table 3

Supplementary table 4

## Data Availability

The datasets generated during and/or analysed during the current study are available in the Mendeley Data repository, https://data.mendeley.com/datasets/kxx8kjcyfx/1.
