# Supplementary material for: Dendritiform immune cells with reduced antigen-capture capacity persist in the cornea during the asymptomatic phase of allergic conjunctivitis
Source: Eye (Lond). 2023 Feb 6;37(13):2768–75. doi: 10.1038/s41433-023-02413-2 (PMC10482935; doi:10.1038/s41433-023-02413-2)
Supplement: Supplementary file 1 — Supplementary table 1 [file 41433_2023_2413_MOESM1_ESM.docx]

Supplementary table 1: Skin prick test results of 20 allergic participants during the active phase of allergy. +ve: Skin prick test positive (+ve); DP: Dermatophagoides pteronyssinus. Gray cells indicate negative results (wheal size below 3 mm diameter).

| **Participant ID** | **Dust mites** | | **Grass Pollen** | | | **Tree Pollen** | **Plant Pollen** | **Mould** | **Animal Dander** | |
| --- | --- | --- | --- | --- | --- | --- | --- | --- | --- | --- |
|  | DP | Farinae | Rye | Bermuda | Paspalum | Cypress | Plantain | Alternaria sp | Cat | Dog |
| 1 | +ve | +ve |  |  |  |  | +ve | +ve | +ve |  |
| 2 | +ve | +ve |  | +ve | +ve |  |  |  | +ve |  |
| 3 | +ve | +ve |  | +ve | +ve |  |  |  |  |  |
| 4 |  | +ve |  |  |  | +ve |  |  |  |  |
| 5 | +ve | +ve | +ve | +ve | +ve | +ve | +ve |  | +ve |  |
| 6 |  |  | +ve | +ve | +ve |  | +ve | +ve |  |  |
| 7 | +ve | +ve |  |  |  |  |  |  |  |  |
| 8 |  | +ve | +ve |  |  |  |  |  |  |  |
| 9 | +ve | +ve | +ve | +ve | +ve | +ve | +ve |  |  |  |
| 10 |  | +ve | +ve | +ve | +ve |  | +ve |  |  |  |
| 11 | +ve | +ve |  |  |  |  |  |  |  |  |
| 12 |  |  | +ve | +ve | +ve |  |  |  |  |  |
| 13 |  |  | +ve | +ve |  | +ve |  |  | +ve | +ve |
| 14 | +ve | +ve | +ve | +ve | +ve | +ve | +ve |  | +ve | +ve |
| 15 |  | +ve |  |  | +ve |  |  |  |  |  |
| 16 |  |  | +ve | +ve | +ve |  |  |  |  |  |
| 17 | +ve | +ve | +ve | +ve |  | +ve | +ve | +ve | +ve | +ve |
| 18 | +ve | +ve |  |  |  |  |  |  |  |  |
| 19 | +ve | +ve | +ve | +ve | +ve |  | +ve | +ve |  | +ve |
| 20 | +ve | +ve |  | +ve |  |  |  |  |  |  |
