# Supplementary material for: Dendritiform immune cells with reduced antigen-capture capacity persist in the cornea during the asymptomatic phase of allergic conjunctivitis
Source: Eye (Lond). 2023 Feb 6;37(13):2768–75. doi: 10.1038/s41433-023-02413-2 (PMC10482935; doi:10.1038/s41433-023-02413-2)
Supplement: Supplementary file 2 — Supplementary table 2 [file 41433_2023_2413_MOESM2_ESM.docx]

Supplementary table 2: Dendritic cell density across corneal and conjunctival locations using *in vivo* confocal microscopy in participants during active (n=20) and asymptomatic (n=20) phases of allergic conjunctivitis. Data are expressed as Median (IQR).

| **Location** | **Dendritic cell density (cells/mm^2^)** | | **p-value** |
| --- | --- | --- | --- |
|  | **Active (n=20)** | **Asymptomatic (n=20)** |  |
| Corneal centre | 28.7 (10.3-127.5) | 24.4 (7.8-153.4) | 0.95 |
| Inferior whorl | 25.0 (6.2-87.5) | 28.1 (18.7-96.8) | 0.22 |
| Corneal periphery | 56.9 (20.9-84.7) | 48.7 (21.9-73.4) | 0.88 |
| Corneal limbus | 103.1 (66.9-149.1) | 75.6 (41.9-136.9) | 0.47 |
| Bulbar conjunctiva | 12.5 (0-45.3) | 0 (0-16.6) | ***0.01*** |
