# Supplementary material for: Dendritiform immune cells with reduced antigen-capture capacity persist in the cornea during the asymptomatic phase of allergic conjunctivitis
Source: Eye (Lond). 2023 Feb 6;37(13):2768–75. doi: 10.1038/s41433-023-02413-2 (PMC10482935; doi:10.1038/s41433-023-02413-2)
Supplement: Supplementary file 3 — Supplementary table 3 [file 41433_2023_2413_MOESM3_ESM.docx]

Supplementary table 3: Association between dendritic cell density/ morphology and ocular surface symptoms/signs examined using Spearman correlation and Mann-Whitney U test as appropriate in all participants. All 5 locations were included, *therefore* the level of significance set at p<0.01 (adjusted for multiple comparisons).

|  | DC Density | DC morphology | | | |
| --- | --- | --- | --- | --- | --- |
|  |  | Cell body size | Presence of dendrites | Presence of long dendrites | Presence of thick dendrites |
| Dryness | rho=-0.09  p=0.15 | rho=0.07  p=0.40 | p=0.70 | p=0.80 | p=0.40 |
| Itchiness | rho=0.003  p=0.96 | rho=0.06  p=0.45 | p=0.95 | p=0.25 | p=0.80 |
| Burning | rho=-0.07  p=0.35 | rho=0.03  p=0.65 | p=0.55 | p=0.20 | p=0.90 |
| Stinging | rho=0.10  p=0.85 | rho=0.02  p=0.80 | p=0.95 | p=0.70 | p=0.50 |
| Watering | rho=-0.06  p=0.35 | rho=-0.08  p=0.25 | p=0.60 | p=0.40 | p=0.40 |
| Redness | rho=0.003  p=0.95 | rho=0.02  p=0.80 | p=0.80 | p=0.25 | p=0.30 |
| A need to rub eyes | rho=0.04  p=0.35 | rho=0.08  p=0.30 | p=0.25 | p=0.013 | p=0.60 |
| AUAQ, Total symptom score | rho=-0.07  p=0.35 | rho=0.01  p=0.98 | p=0.80 | p=0.45 | p=0.95 |
| OSDI | rho=-0.05  p=0.50 | rho=0.06  p=0.45 | p=0.90 | p=0.60 | p=0.80 |
| DEQ-5 | rho=-0.08  p=0.25 | rho=0.004  p=0.95 | p=0.50 | p=0.70 | p=0.15 |
| Limbal redness | rho=0.004  p=0.95 | ***rho=0.26***  ***p=0.001*** | p=0.09 | ***p=0.001*** | p=0.15 |
| Bulbar redness | rho=0.04  p=0.60 | ***rho=0.24***  ***p=0.002*** | p=0.15 | ***p=0.001*** | p=0.25 |
| Palpebral redness | rho=0.03  p=0.65 | ***rho=0.27***  ***p=0.001*** | p=0.05 | ***p=0.001*** | p=0.40 |
| Bulbar conjunctival chemosis | rho=0.001  p=0.98 | rho=0.17  p=0.03 | p=0.40 | p=0.011 | p=0.30 |
| Palpebral conjunctival follicle | rho=0.05  p=0.51 | rho=0.17  p=0.03 | p=0.80 | p=0.10 | p=0.70 |
| Conjunctival staining-Nasal | rho=0.05  p=0.45 | rho=0.15  p=0.05 | p=0.25 | p=0.15 | p=0.03 |
| Conjunctival staining-Temporal | rho=0.16  p=0.02 | ***rho=0.21***  ***p=0.006*** | p=0.08 | p=0.02 | p=0.04 |
| Non-invasive Tear film Break-Up Time | rho=-0.08  p=0.30 | rho=-0.17  p=0.03 | p=0.45 | p=0.02 | p=0.25 |
