# Supplementary material for: Dendritiform immune cells with reduced antigen-capture capacity persist in the cornea during the asymptomatic phase of allergic conjunctivitis
Source: Eye (Lond). 2023 Feb 6;37(13):2768–75. doi: 10.1038/s41433-023-02413-2 (PMC10482935; doi:10.1038/s41433-023-02413-2)
Supplement: Supplementary file 4 — Supplementary table 4 [file 41433_2023_2413_MOESM4_ESM.docx]

Supplementary table 4: Difference in conjunctival dendritic cell (DC) density between active and asymptomatic phase of allergy and the differences in symptoms/ signs between active and asymptomatic phase of allergy.

| Differences in symptoms/ signs between the active and asympotmatic phase | Correlation with difference in conjunctival DC density between active and asympotmatic phase |
| --- | --- |
| AUAQ, Total symptom score | rho=-0.15, p=0.66 |
| OSDI | rho=0.29, p=0.33 |
| DEQ-5 | rho=0.15, p=0.61 |
| Limbal redness | rho=0.14, p=0.65 |
| Bulbar redness | rho=-0.30, p=0.32 |
| Palpebral redness | rho=0.004, p=0.98 |
| Non-invasive Tear film Break-Up Time | rho=-0.26, p=0.50 |
